# Identification of MicroRNA-Target Gene-Transcription Factor Regulatory Networks in Colorectal Adenoma Using Microarray Expression Data

**DOI:** 10.3389/fgene.2020.00463

**Published:** 2020-05-19

**Authors:** Yadong Gao, Shenglai Zhang, Yan Zhang, Junbo Qian

**Affiliations:** ^1^Department of Gastroenterology, The Second Affiliated Hospital of Nantong University, Nantong, China; ^2^Department of Gastroenterology, The First People’s Hospital of Nantong, Nantong, China

**Keywords:** colorectal adenoma, differentially expressed miRNAs, differentially expressed genes, transcription factor, regulatory network, bioinformatic

## Abstract

**Objective:**

The aim of the study was to find the key genes, microRNAs (miRNAs) and transcription factors (TFs) and construct miRNA-target gene-TF regulatory networks to investigate the underlying molecular mechanism in colorectal adenoma (CRA).

**Methods:**

Four mRNA expression datasets and one miRNA expression dataset were downloaded from Gene Expression Omnibus (GEO) database. Differentially expressed miRNAs (DEMs) and differentially expressed genes (DEGs) were identified between CRA and normal samples. Moreover, functional enrichment analysis for DEGs was carried out utilizing the Cytoscape-plugin, known as ClueGO. These DEGs were mapped to STRING database to construct a protein-protein interaction (PPI) network. Then, a miRNA-target gene regulatory network was established to screen key DEMs. In addition, similar workflow of the analyses were also performed comparing the CRC samples with CRA ones to screen key DEMs. Finally, miRNA-target gene-TF regulatory networks were constructed for these key DEMs using iRegulon plug-in in Cytoscape.

**Results:**

We identified 514 DEGs and 167 DEMs in CRA samples compared to healthy samples. Functional enrichment analysis revealed that these DEGs were significantly enriched in several terms and pathways, such as regulation of cell migration and bile secretion pathway. A PPI network was constructed including 325 nodes as well as 890 edges. A total of 59 DEGs and 65 DEMs were identified in CRC samples compared to CRA ones. In addition, Two key DEMs in CRA samples compared to healthy samples were identified, such as hsa-miR-34a and hsa-miR-96. One key DEM, hsa-miR-29c, which was identified when we compared the differentially expressed molecules found in the comparison CRA versus normal samples to the ones obtained in the comparison CRC versus CRA, was also identified in CRC samples compared to CRA ones. The miRNA-target gene-TF regulatory networks for these key miRNAs included two TFs, one TF and five TFs, respectively.

**Conclusion:**

These identified key genes, miRNA, TFs and miRNA-target gene-TF regulatory networks associated with CRA, to a certain degree, may provide some hints to enable us to better understand the underlying pathogenesis of CRA.

## Introduction

Colorectal adenoma (CRA) is defined as non-cancerous lesion of the large intestinal epithelium. It is commonly acknowledged that CRA is a precancerous condition and may progress to colorectal cancer (CRC) that is a common reason of cancer death worldwide, with the unknown etiology and pathogenesis (Fearon, 2010; Kim et al., 2020). In spite of certain progress recently, the understanding of underlying biomolecules of CRA is rather rudimentary and the process of screening these biomolecules is still continuing.

Previously, many published reports focused on the carcinogenesis of CRA have been performed. For instance, *KRAS* and *TP53* mutations have been reported to participate in the adenoma-carcinoma sequence (Fearon, 2010). A recent study also demonstrated that 5 miRNA ratios were significantly up-regulated in serum samples from patients with CRC compared with the ones from patients with CRA (Zhang et al., 2018). Furthermore, a few studies regarding CRA pertained to its pathogenesis. Joosten et al. (2017) demonstrated that HGF receptor signaling regulated the formation of CRA. Notwithstanding studies of differentially expressed genes (DEGs) and differentially expressed miRNAs (DEMs) have been carried out in the last few years and some of their biological function have been elucidated, the detailed mechanisms associated with the pathogenesis of CRA still remain poorly understood on account of a limited number of identified genetic alterations and unknown interactions among DEGs and DEMs.

In the present study, we chose four mRNA expression profiles (GSE31905, GSE4183, GSE37364, and GSE41657) and one miRNA expression dataset (GSE41655), which were downloaded from the GEO database, in order to identify DEGs and DEMs found in the comparison CRA versus normal samples and in the comparison CRC versus CRA samples. Subsequently, miRNA-target gene network analysis was carried out. For further study, transcription factors (TFs) in relation to the key DEMs from the interaction network were identified. The aim of the present research was to identify key genes, miRNAs and TFs of CRA and construct the miRNA-target gene-TF regulatory networks to explore the underlying molecular mechanism using bioinformatic methods.

## Materials and Methods

### Microarray Data

In order to identify key genes and miRNAs of CRA and construct the miRNA-target gene-TF regulatory networks related to CRA, we used “CRA” as keywords to search for genome-wide expression studies in GEO database. Only datasets which included normal samples, CRA samples and CRC samples were the first choice for inclusion. In addition, the types of studies were non-coding RNA profiling by array or expression profiling by array. Finally, the datasets of GSE31905, GSE4183, GSE37364, GSE41657, and GSE41655 were downloaded from the GEO database. The author, year, platform and the proportions of CRC, CRA and normal samples in each dataset were extracted and evaluated. Details of expression profile datasets were presented in Table 1.

**TABLE 1:** Characteristics of mRNA and miRNA expression profiles of colorectal adenoma.

**Author, year**	**Accession^a^**	**Platform**	**Samples(normal/CRA samples/CRC)**
Anders et al., 2013	GSE31905	GPL6480	7/5/55
Gyorffy et al., 2009	GSE4183	GPL570	8/15/15
Galamb et al., 2012	GSE37364	GPL570	38/29/27
Li et al., 2015	GSE41657	GPL6480	12/51/25
Li et al., 2015	GSE41655	GPL11487	15/59/33

*^*a*^The first four datasets are mRNA expression profiles and the last one is miRNA expression profiles. miRNA, microRNA; CRA, colorectal adenoma; CRC, colorectal cancer.*

### Analysis of DEMs and DEGs

GEO2R^1^ is an interactive web-based tool, which is able to compare two data sets in a GEO series. We used data which had been normalized to detect DEGs and DEMs by using GEO2R. Among these dataset, the dataset of GSE31905 was pre-processed using the agilent algorithm and was normalized per chip to the 50th percentile. The dataset of GSE4183 was normalized by applying RMA method. The dataset of GSE37364 was normalized by applying the function mas5 from the bioconductor affy package. The dataset of GSE41657 was normalized per chip to the 50th percentile by using the GeneSpringGX software v11.5. For the dataset of GSE41655, quantile normalization was performed by R project. For purpose of reducing the false positive rate, the *p*-value was adjusted by utilizing Benjamini–Hochberg false discovery rate (FDR) method (Reiner et al., 2003). The multiple *t*-test was utilized to detect statistically significant molecules at the same time with FDR correction. The probes with no gene annotation were removed. Genes with an adjusted *p* < 0.05 as well as | log_2_ fold change (FC)| > 1 were chosen as DEGs. DEMs were screened with the thresholds of adjusted *p*-value < 0.05 as well as |log_2_ FC| > 0.5.

### Functional and Pathway Enrichment Analysis

In order to have an in-depth understanding of biological significance of DEGs, Gene Ontology (GO) and Kyoto Encyclopedia of Genes and Genomes (KEGG) pathway enrichment analyses were carried out by utilizing the Cytoscape software v3.6.1 and Cytoscape-plugin, known as ClueGO v2.5.1 (Bindea et al., 2009). ClueGO plugin v2.5.1, which improves biological interpretation of a number of gene lists, can find out and functionally categorize significant GO terms and KEGG pathways. A *p*-value < 0.05 was selected as a cut-off criterion for GO terms as well as KEGG pathway enrichment analysis.

### PPI Network Construction and Analysis of Modules

As an online software containing co-expression, co-occurrence as well as protein–protein interaction (PPI) information, the STRING database 10.5^2^ provides an assessment and integration of PPI, including physical as well as functional connections. In this research, the previously identified DEGs were mapped to STRING database in order to analyze the PPI of DEGs. Combined score ≥ 0.4 was set as the threshold. In addition, Cytoscape was utilized to construct the protein interaction network. The Cytoscape-plugin known as CytoNCA v2.1.6 (Tang et al., 2015) was used to calculate the score of gene nodes by using three centrality methods (Degree Centrality, Betweenness Centrality, and Closeness Centrality; Cukierski and Foran, 2008; Opsahl et al., 2010; Du et al., 2015). Subsequently, a Cytoscape-plugin, known as Molecular Complex Detection (MCODE) v1.5.1 (Bader and Hogue, 2003), was utilized to construct sub-networks with degree cut-off = 2, max. depth = 100, node score cut-off = 0.2 and k-Core = 2. Moreover, GO terms as well as KEGG pathways analyses of DEGs in key module were carried out by utilizing ClueGO plugin v2.5.1.

### Prediction of Target Genes for DEMs

The potential candidate target genes of the DEMs from GSE41655 were predicted with miRWalk 2.0 (Dweep and Gretz, 2015). The online tools of miRWalk 2.0 based on 6 bioinformatic algorithms [miRWalk (Dweep and Gretz, 2015), Pictar2 (Krek et al., 2005), PITA (Kertesz et al., 2007), RNA22 (Miranda et al., 2006), RNAhybrid (Kruger and Rehmsmeier, 2006), and Targetscan (Lewis et al., 2005)] were utilized to predict the potential candidate target genes of aberrant miRNAs. Only the target genes which were common in the prediction of all of the above algorithms were screened out. Then, overlaps among these target genes and common DEGs in the four gene expression profile datasets were obtained for further analysis. Afterward, the regulatory network between the overlaps and DEMs was constructed, which was visualized by utilizing Cytoscape. Moreover, the network topology was analyzed using CytoNCA with the three algorithms mentioned above in order to identify key DEMs.

### Construction of the miRNA-Target Gene-Transcription Factor (TF) Regulatory Network

miRNAs and TFs play the part of trans-regulators which controlled gene regulatory networks in a dependent or independent way (Zhao et al., 2016). Hence, understanding of miRNAs function may be deeper in the context of regulatory interactions between TFs and miRNAs. In this study, potential TFs in relation to the key DEMs were predicted utilizing iRegulon plug-in v1.3 (Janky et al., 2014) with enrichment score threshold = 5.0, ROC threshold for AUC calculation = 0.05, rank threshold = 5000, minimum identity between orthologous genes = 0.05 and maximum FDR on motif similarity = 0.001 in Cytoscape, and the miRNAs-target gene-TF regulatory networks were constructed.

## Results

### Colorectal Adenoma Versus Normal

#### Screening of DEGs and DEMs

**FIGURE 1:**
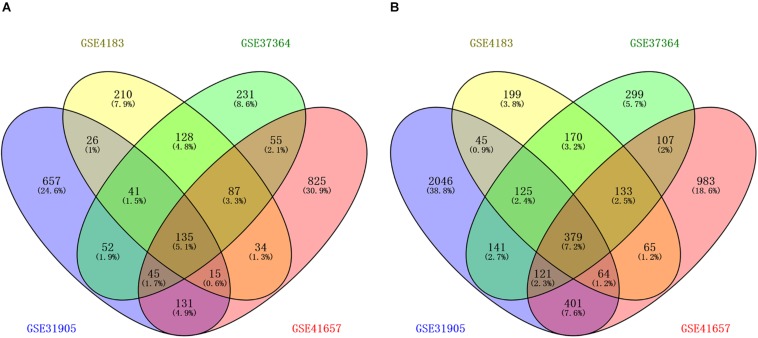
Identification of differentially expressed genes in four mRNA expression profile datasets (GSE31905, GSE4183, GSE37364, and GSE41657). **(A)** The overlapping up-regulated genes in four gene expression profiles; **(B)** the overlapping down-regulated genes in four gene expression profiles. Each circle depicts the number of expressed genes. Overlapping expressed genes shared more than one dataset is represented in the area of intersection between 2 circles.

The workflow of the methodology used in our study was shown in the Supplementary Figure 1. A total of four mRNAs and one miRNA expression profile datasets, which included 168 CRA and 87 healthy samples, were obtained from GEO database. Four gene expression profiles (GEO accession nos. GSE31905, GSE4183, GSE37364, and GSE41657) identified 1102 upregulated genes, 676 upregulated genes, 774 upregulated genes, and 1327 upregulated genes respectively, and identified 3322 downregulated genes, 1180 downregulated genes, 1475 downregulated genes, and 2253 downregulated genes, respectively. The overlapping up-regulated genes in four gene expression profiles and overlapping down-regulated genes in four gene expression profiles were considered as common DEGs (Figure 1 and Supplementary Table 1). Finally, 514 common DEGs were screened, including 135 upregulated genes as well as 379 downregulated genes in CRA samples in comparison with normal colorectal samples. In addition, the miRNA expression profile (GSE41655) identified 167 DEMs, including 61 upregulated miRNAs as well as 106 downregulated miRNAs.

#### Functional and Pathway Enrichment Analysis

**FIGURE 2:**
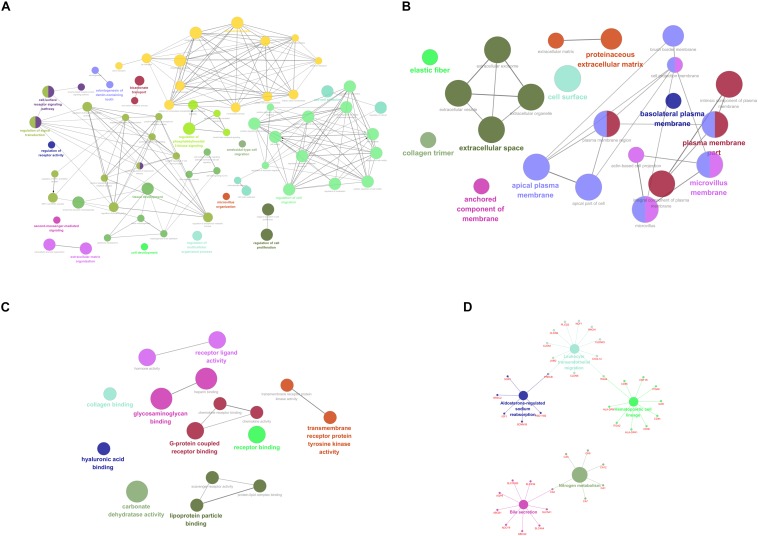
Functionally enriched GO terms and KEGG pathways analysis of DEGs in colorectal adenoma. Each group is represented by only the label of the most significant term/pathway. A grouped network of functionally enriched genes generated for the DEGs using ClueGO. The node size indicates the term/pathway enrichment significance. Functionally associated groups partially overlap. **(A)** BP-associated category; **(B)** CC-associated category; **(C)** MF-associated category; **(D)** KEGG pathways. DEGs, differentially expressed genes; GO, Gene Ontology (GO); BP, biological processes; CC, cellular component; MF, molecular function; KEGG, Kyoto Encyclopedia of Genes and Genomes.

For purpose of gaining insights into the function and mechanism of these DEGs, GO terms including biological process (BP), cellular component (CC), and molecular function (MF), as well as KEGG pathways were enriched utilizing ClueGO plugin. With *p*-value < 0.05 as the threshold, significantly enriched GO terms as well as pathways of the identified DEGs were present in Figure 2. In the BP-associated category, the most significantly enriched GO terms were regulation of cell migration (GO:0030334) with *p*-value = 4.67E-14, regulation of cell motility (GO:2000145) with *p*-value = 1.05E-12, regulation of locomotion (GO:0040012) with *p*-value = 3.98E-12, regulation of cellular component movement (GO:0051270) with *p*-value = 2.37E-11 and cell surface receptor signaling pathway with *p*-value = 6.73E-11. Moreover, the most significantly enriched GO terms were extracellular space (GO:0005615) with *p*-value = 2.25E-11, plasma membrane part (GO:0044459) with *p*-value = 2.00E-08, microvillus membrane (GO:0031528) with *p*-value = 9.38E-08, intrinsic component of plasma membrane (GO:0031226) with *p*-value = 1.38E-07 and apical plasma membrane (GO:0016324) with *p*-value = 1.86E-07 in the cell component (CC) -associated category. In addition, these identified DEGs were significantly enriched in glycosaminoglycan binding (GO:0005539) with *p*-value = 2.67E-11, heparin binding (GO:0008201) with *p*-value = 8.42E-09, carbonate dehydratase activity (GO:0004089) with *p*-value = 8.81E-07, G-protein coupled receptor binding (GO:0001664) with *p*-value = 1.06E-05 and receptor ligand activity (GO:0048018) with *p*-value = 1.09E-05 in the CC-associated category. Moreover, the identified DEGs were mainly enriched in nitrogen metabolism (KEGG:00910) with *p*-value = 4.30E-06, bile secretion (KEGG:04976) with *p*-value = 1.47E-04, Leukocyte transendothelial migration (KEGG:04670) with *p*-value = 2.81E-04, hematopoietic cell lineage (KEGG:04670) with *p*-value = 3.59E-04 and aldosterone-regulated sodium reabsorption (KEGG:04960) with *p*-value = 5.05E-04.

#### PPI Network Construction and Analysis of Modules

**FIGURE 3:**
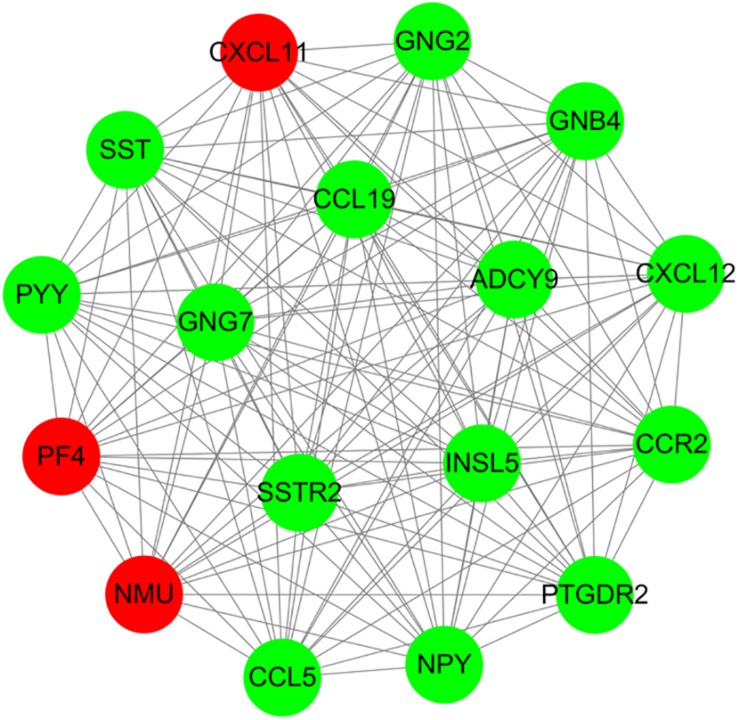
A sub-network module with the highest score in PPI network conducted based on 514 DEGs between CRA and normal tissues. Red nodes and green nodes indicate upregulated and downregulated genes, respectively. PPI, protein–protein interaction; DEGs, differentially expressed genes; CRA, colorectal adenoma.

For 514 DEGs between CRA and normal tissues, a PPI network was constructed, including 325 nodes as well as 890 edges (Supplementary Figure 2). Top 20 nodes with high degrees were identified using a network topology analysis. Then, 13 common genes among these top 20 nodes were screened as hub genes based on the three centrality methods (Table 2), such as PH domain leucine-rich repeat protein phosphatase 2 (*PHLPP2*), *V*-myc myelocytomatosis viral oncogene homolog (*MYC*), insulin-like growth factor 1 (*IGF1*), CD44 molecule (*CD44*), guanine nucleotide binding protein 4 (*GNB4*), chemokine ligand 12 (*CXCL12*), colony stimulating factor 1 receptor (*CSF1R*), adenylate cyclase 9 (*ADCY9*), chemokine ligand 5 (*CCL5*), glucagon (*GCG*), decorin (*DCN*), integrin, alpha 2 (*ITGA2*) and apolipoprotein E (*APOE*). Next, the sub-network module analysis was conducted and a total of 16 cluster modules were obtained. The top module with the highest score (score: seventeen) contained 17 nodes and 136 edges (Figure 3).

**TABLE 2:** Top 20 nodes of DEGs in PPI network conducted based on 514 DEGs between CRA tissues and normal tissues.

**Gene**	**Degree**	**Gene**	**Betweenness**	**Gene**	**Closeness**
PHLPP2	44	PHLPP2	19635.186	MYC	0.03199684
MYC	39	MYC	12719.717	IGF1	0.031952664
IGF1	37	CD44	9873.984	PHLPP2	0.031896044
CD44	33	IGF1	9854.683	CXCL12	0.03188035
GNB4	33	CSF1R	7981.7646	CD44	0.031858407
CXCL12	30	DCN	7626.397	GNB4	0.031858407
SST	29	GNB4	6612.885	MET	0.031808365
CSF1R	26	PRKCB	4880.521	CSF1R	0.031805243
ADCY9	26	APOE	4200.9272	CCL5	0.031789638
GNG2	26	CCND1	3927.6084	APOE	0.031739812
GNG7	25	MET	3911.5237	SST	0.031739812
CCR2	24	CXCL12	3789.8855	GCG	0.031714957
NPY	24	GCG	3778.8518	DCN	0.031702545
CCL5	24	PRKAR2B	3672.393	CCR2	0.03168704
GCG	23	ITGA2	3342.6714	ADCY9	0.031683944
DCN	21	CCL5	2937.6538	ITGA2	0.031656083
CCL19	21	ADCY9	2931.6262	GNG2	0.031646807
PF4	21	CACNA1C	2881.468	VIP	0.031643715
ITGA2	20	KLRD1	2849.4792	CCND1	0.031631358
APOE	20	LGR5	2745.487	KLF4	0.03161901

*Degree, results of degree centrality algorithm; betweenness, results of betweenness centrality algorithm; closeness, results of Closeness centrality algorithm; DEG, differentially expressed genes; PPI, protein–protein interaction.*

Furthermore, all DEGs in the top module conducted GO functional enrichment as well as KEGG pathway analyses. Among these DEGs, the genes in the module were significantly enriched in 35 GO terms, including chemokine receptor binding (*P* = 1.37E-11), chemokine-mediated signaling pathway (*P* = 6.34E-11), positive regulation of leukocyte chemotaxis (*P* = 1.77E-10), chemokine activity (*P* = 6.16E-10), and regulation of leukocyte chemotaxis (*P* = 7.53E-10). Additionally, these DEGs were categorized into 5 significant KEGG pathways, such as chemokine signaling pathway (*P* = 8.06E-14), GABAergic synapse (*P* = 8.06E-14), orphine addiction (*P* = 2.08E-05), circadian entrainment (*P* = 2.57E-05), and gastric acid secretion (*P* = 3.52E-04).

#### Prediction of Target Genes for DEMs

**FIGURE 4:**
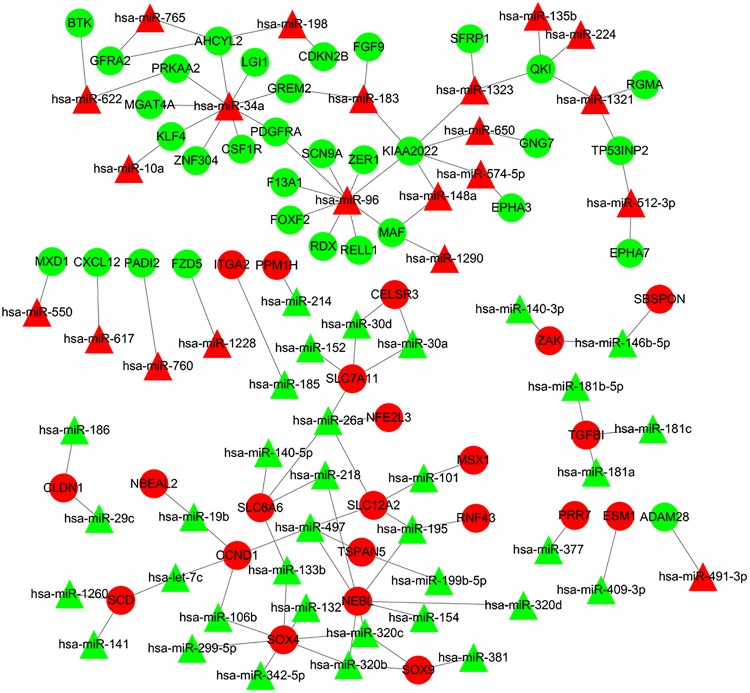
Regulatory network of DEMs and their target DEGs in the comparison CRA samples versus normal samples. Red nodes and green nodes indicate upregulated genes/miRNAs and downregulated genes/miRNAs, respectively. Triangles represent DEMs and circles represent target DEGs. miRNAs, microRNAs; DEMs, differentially expressed miRNAs; DEGs, differentially expressed genes; CRA, colorectal adenoma.

The online tools of miRWalk 2.0 based on 6 bioinformatic algorithms (miRWalk2.0, Pictar2, PITA, RNA22v2, RNAhybrid2.1, and Targetscan6.2) were utilized to predict the potential candidate target genes of aberrant miRNAs. Only the target genes which were common in the prediction of all of the above algorithms were screened out. A total of 2231 target genes were obtained. Subsequently, 80 overlaps were identified among the common DEGs in four mRNA expression profile datasets and target genes. After removal of these overlaps whose expression levels were positively correlated with that of miRNAs, the regulatory network between the overlaps and DEMs was constructed. As showed in Figure 4, the regulatory network contained 111 nodes and 106 edges, including 21 upregulated miRNAs, 35 downregulated miRNAs, 22 upregulated target genes, and 33 downgraded target genes. The top 10 nodes with high degrees were identified based on the analysis of network topology. We gained 2 common DEMs (hsa-miR-34a and hsa-miR-96) among these top 10 nodes. All these common DEMs were upregulated, including hsa-miR-34a predicted to regulate 9 genes and hsa-miR-96 predicted to regulate 9 genes (Supplementary Table 2).

### Construction of the miRNA-Target Gene-Transcription Factor (TF) Regulatory Network

**FIGURE 5:**
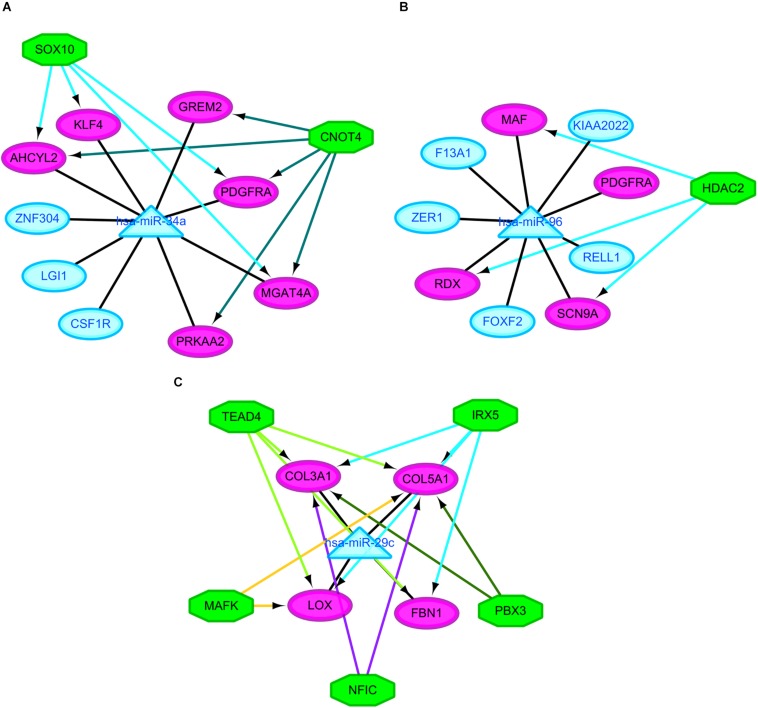
Regulatory networks of the key miRNAs, target genes and transcription factors. **(A)** Network of hsa-miR-34a and 2 transcription factors; **(B)** network of hsa-miR-96 and one transcription factor; **(C)** network of hsa-miR-29c and 5 transcription factors. Green octagons indicate transcription factors. Purple circles indicate target genes regulated by key miRNAs and transcription factors, blue circles indicate target genes regulated by key miRNAs and triangles indicate key miRNAs. miRNA, microRNAs.

It was hypothesized that miRNAs with high degrees in the regulatory network might play a significant role in CRA pathogenesis. Therefore, the two common miRNAs (hsa-miR-34a and hsa-miR-96) were identified as key miRNAs. For further elucidation of these key miRNAs function, the miRNA-common target gene-TF regulatory networks for these key miRNAs were constructed, respectively. As showed in Figure 5 and Supplementary Table 2, the regulatory network for hsa-miR-34a and hsa-miR-96 included two TFs and one TF, respectively.

### Colorectal Cancer Versus Colorectal Adenoma

The similar workflow of the analyses also was performed comparing the CRC samples with CRA ones (Supplementary Figure 1). A total of 59 common DEGs were screened, including 51 upregulated genes as well as 8 downgraded genes in CRC samples in comparison with CRA samples. In addition, A total of 65 DEMs, including 43 upregulated miRNAs as well as 22 downgraded miRNAs, were screened. The online tools of miRWalk 2.0 based on 6 bioinformatic algorithms (miRWalk2.0, Pictar2, PITA, RNA22v2, RNAhybrid2.1, and Targetscan6.2) were utilized to predict the potential candidate target genes of aberrant miRNAs. A total of 1500 target genes were obtained. Subsequently, 10 overlaps were identified among the common DEGs in four mRNA expression profile datasets and target genes. After removal of differentially expressed target genes whose expression levels were positively correlated with that of miRNAs, 16 miRNA-gene pairs were identified. The regulatory network based on the miRNA-gene pairs was constructed. As seen in Supplementary Figure 3, the regulatory network contained 19 nodes and 16 edges, including 11 downregulated miRNAs and 8 upregulated target genes. The top ten nodes with high degrees were identified based on the analysis of network topology. We gained 7 common DEMs (hsa-miR-29c, hsa-miR-30e, hsa-miR-30d, hsa-miR-200a, hsa-miR-194, hsa-miR-141, and hsa-miR-377) among these top 10 nodes. All these common DEMs were down-regulated, including hsa-miR-29c predicted to regulate 4 genes, hsa-miR-30e predicted to regulate 2 genes, hsa-miR-30d predicted to regulate 2 genes, hsa-miR-200a predicted to regulate 2 genes, hsa-miR-194 predicted to regulate 1 genes, hsa-miR-141 predicted to regulate 1 genes, hsa-miR-377 predicted to regulate 1 genes. In addition, we compared the differentially expressed molecules found in the comparison CRA versus normal samples to the ones obtained in the comparison CRC versus CRA. After comparison, 9 common DEMs such as downregulated hsa-miR-29c were found. However, no common differentially DEGs were screened out. Finally, miRNA-common target gene-TF regulatory network for the key miRNA hsa-miR-29c was constructed. As showed in Figure 5 and Supplementary Table 2, the regulatory network for hsa-miR-29c included 5 TFs.

## Discussion

Recently, CRC is the third major reason of cancer-related mortality. Although the death rate of cancer has been decreasing in the recent past because of improved treatment and early diagnosis, many people succumb to CRC every year yet. It is well accepted that CRA is the major precursor of CRC. Investigation into mRNA and miRNA expression changes in the progression of CRA can provide insights into the molecular mechanism involved in CRA and help to facilitate early diagnosis, treatment and prevention of CRA. Previous studies have examined expression profiles involved in CRA to identify DEGs and DEMs. Nevertheless, discrepant results were gained owing to different platform and control selection. Due to limitations on the comparative analysis of DEGs as well as DEMs in independent studies, there are still questions about the interactions among DEGs and DEMs involved in CRA. Additionally, the synergistic effects of TFs as well as miRNAs in the context of genetic regulatory networks are largely unknown. As far as we know, this is the first attempt to reveal interactions between DEGs and DEMs regarding to CRA in miRNAs and mRNA expression profiling datasets and construct the miRNA-target gene-TF regulatory networks. Identification and analysis of CRA-associated genes, miRNAs and TFs may reveal the potential pathogenesis of CRA on the level of molecules and help to facilitate in CRA diagnosis and treatment.

In this study, a total of 514 common DEGs were identified among these four mRNA expression profiling datasets, consisting of 135 upregulated genes and 379 downgraded genes in CRA sample tissues in comparison with healthy colorectal tissues. For purpose of understanding fully the function and mechanism of these DEGs, we conducted GO categories and pathway terms enrichment analyses utilizing ClueGO plugin. We found that 73 GO_BP, 27 GO_CC as well as 16 GO_MF terms were involved. Functional enrichment analysis revealed that the DEGs were related to cell activity including regulation of cell migration, motility and proliferation, which have previously been shown to relevant to cancer progression (Sawai et al., 2005). As the precancerous of colorectal carcinoma, the potential pathogenesis of CRA may be associated with cell activity. Pathway enrichment analysis indicated that these identified genes were significantly enriched in five pathways, such as bile secretion. Previous study reported that bile acids played a crucial role in the etiology of CRC, and there was an increase of the secondary bile acids in the plasma of patients with CRA (Cao et al., 2014). Nine DEGs were related to the pathway, including 2 upregulated genes and 7 downgraded genes. Among these genes, certain genes are members of ATP binding cassette subfamily including *ABCB1* as well as *ABCG2*, which have previously been demonstrated to bear some relationship to the progression of CRA. Andersen et al. (2015) found that *ABCB1* and *ABCG2* mRNA levels were significantly lower in adenomas in comparison with tissue from healthy individuals, consistent with the results of this present study. It was noteworthy that some researchers noted that the unconjugated secondary bile acids might promote stemness in colonic epithelial cells and induced the production of cancer stem cells, which was associated with the development of CRA. The proportion of cancer stem cells increased gradually together with the increased levels of several cancer stem cells markers including *ABCB1* and *ABCG2* (Farhana et al., 2016), which was not completely consistent with the results of this study. Therefore, the functions of *ABCB1* and *ABCG2* involved in CRA development should be pursued.

In addition, we identified 13 high degree genes by constructing the PPI, including 3 up-regulated genes as well as 10 down-regulated genes. Of these DEGs, certain genes have previously been reported to be related to the genesis and development in CRA. A previous study demonstrated that *GCG* was constantly down-regulated in adenomas in comparison with the gene expression level in normal colorectal mucosa (Wu et al., 2018). Hashimoto et al. (2017) reported that *MYC* overexpression and nuclear β-catenin accumulation, which indicated that the WNT signal pathway was activated, were identified in the majority of sessile serrated adenoma/polyps with dysplasia. These data suggest that these genes may serve key functions in the histogenesis and development of CRA. Furthermore, the module enrichment analysis showed that the hub module was significantly enriched in chemokine receptor binding and chemokine signaling pathway, and the majority of the nodes in the hub module were chemokines or their receptors, such as *CCL19*, *CCL5*, and *CXCL12*. Chemokines were small proteins that specifically responded to proinflammatory stimuli, and was involved in the pathogenesis of some diseases including CRA and CRC (Johdi et al., 2017). McLean et al. (2011) demonstrated that some inflammatory cytokine genes, such as *CCL5* and *CCL19* were dysregulated in adenomas. Furthermore, Oliveira Frick et al. (2011) found that *CXCL12* showed a dramatical down-regulation in CRA and CRC samples, while the cognate receptor *CXCR4* were dramatically upregulated in tumor tissues compared with corresponding non-affected tissues. These results were accordant to our findings, which strongly indicated a possible link between these chemokines expression and the induction of CRA. Therefore, it may be hypothesized that inflammation promoted adenoma formation and cancerization. The detailed function of these chemokines and potential mechanistic pathways requires further investigation.

miRNAs are non-coding RNA molecules which serve a crucial role in regulating a spectrum of basic cellular processes and they may induce RNA-silencing and work as post-DNA transcription regulators (Catalanotto et al., 2016). Previous researches have reported that the dysregulation of miRNA may be related to numerous diseases, including CRA and CRC (Rottiers and Naar, 2012; Castro et al., 2013; Guo et al., 2016; Zhang et al., 2018). This study screened DEMs in CRA samples in comparison with normal colorectal tissues samples and the potential candidate target genes of the DEMs were predicted. Then, a miRNA-target gene regulatory network was constructed based on the DEMs and the overlaps among the DEGs and potential candidate target genes. Two key DEMs including hsa-miR-34a and hsa-miR-96 which were upregulated in CRA samples in comparison with normal colorectal tissues samples were identified. In the regulatory networks, hsa-miR-34a regulated 9 downregulated genes, such as *KLF4.* MiR-34a was reported to be associated with some metabolic disorders such as obesity, non-alcoholic fatty liver disease and type 2 diabetes, which were implicated in colorectal carcinogenesis (Kong et al., 2011; Rottiers and Naar, 2012; Castro et al., 2013; Nesca et al., 2013; Guo et al., 2016; Uzunlulu et al., 2016). Nesca et al. (2013) demonstrated that the expression of miR-34a was increased in islets of the in diabetic mice, which might be caused by obesity and insulin resistance. They also thought that the miRNA result in increased β-cell apoptosis (Nesca et al., 2013). The expression level of miR-34a was also higher in patients with type 2 diabetes in comparison with healthy controls (Kong et al., 2011). A number of studies reported that patients with non-alcoholic fatty liver disease showed increased hepatic expression of miR-34a (Rottiers and Naar, 2012; Castro et al., 2013; Guo et al., 2016). A potential mechanism for the function of miR-34a in metabolism control might involve a complicated regulatory network of p53, miR-34a and SIRT1. miR-34a regulation of SIRT1 might have an effect on other important metabolic target genes of SIRT1, potentially further result in metabolic disorders, such as obesity (Rottiers and Naar, 2012; Castro et al., 2013). Besides, other potential molecular mechanisms for the function of miR-34a in the metabolism of fat as well as the regulation of energy have also been reported. For example, the expression of target gene, *KLF4*, could be repressed by miR-34a, consequently suppressing anti-inflammatory phenotype macrophage polarization and exacerbating of obesity-related systemic inflammation and metabolic disorder (Pan et al., 2019). These miR-34a-regulated metabolic disorders such as obesity could induce mTORC1 activation (Festuccia, 2020), further contributing to downregulation of miR-29 expression (Slusarz and Pulakat, 2015). Members of the miR-29 family have garnered considerable attention due to their tumor-inhibition function as they were downregulated or silenced in a number of malignancies, such as colorectal cancer (Cristobal et al., 2015; Slusarz and Pulakat, 2015). Our study showed that miR-29c, which regulated 4 upregulated genes such as *CDL3A1*, *LOX*, *COL5A1*, and *FBN1*, was downregulated comparing CRC samples with CRA ones. We also found that miR-29c was downregulated in CRA samples compared to normal samples. These results suggested miR-29c might inhibition the process of initiation and progression of CRA. It should be noted that miR-34a inhibited a lot of cancer-associated processes, such as cell proliferation as well as survival and some researches demonstrated that miR-34a had played a crucial role in suppressing the development of colorectal neoplasms (Siemens et al., 2013; Zhang et al., 2014). Nevertheless, far from the downregulation of miR-34a in colorectal neoplasms, some studies offered exactly opposite data and perspectives (Wang et al., 2012; Hiyoshi et al., 2015). Our study found that miR-34a was upregulated comparing the CRA samples with normal ones, which was consistent with that Slattery’s study (Slattery et al., 2016). A possible explanation to these puzzling results might be two sides of miR-34a. On the one hand, miR-34a as a tumor suppressor played a key role on repressing tumor progression. On the other hand, the p53-dependent upregulation of miR-34a might affect some key metabolic targets, further contributing to downregulation of miR-29 expression by inducing mTORC1 activation, ultimately promoting tumorigenesis. Therefore, suppression of miR-34a might effectively treat metabolic disorders. Metformin, as an agent which has used widely to treat diabetes, could reduce blood sugar and insulin levels and expression of miR-34a (Cifarelli et al., 2015). The agent has certainly been proven to reduced the prevalence as well as number of metachronous CRAs after resection of adenomatous polyp and been related to a significantly lower risk of CRC in type 2 diabetic individuals (Zhang et al., 2011; Higurashi et al., 2016). Besides, ursodeoxycholic acid, which was related to a significant reduction in recurrence of advanced adenoma (Alberts et al., 2005), was demonstrated to suppress expression of miR-34a and be a potentially agent to arrest non-alcoholic fatty liver disease progression (Castro et al., 2013). Based on the above studies and our study, there are reasons to believe that miR-34a and miR-34a-mediated potential metabolic regulators such as *KLF4* can be appropriate candidates for prevention and treatment of CRA patients, especially CRA patients complicated with metabolic disorders. Nevertheless we should be handled with due caution as suppression of miR-34a may cause cancer. Indeed, MRX34, a liposomal miR-34a mimic, has already entered phase I clinical trials in individuals with various malignancies and showed preliminary evidence of antineoplastic activity (Beg et al., 2017). Therefore, the treatment paradigms should be centered on balancing miR-34a level. Similar to miR-34a, another key miRNA which were upregulated in CRA samples in comparison with normal colorectal tissues samples, miR-96, might perform a dual function of tumor suppressor and onco-miRNA (Ress et al., 2015) and promotes the development of hepatic insulin resistance due to saturated fatty acids and obesity (Yang et al., 2016). It suggests that there may be a synergy between the miR-96 and miR-34a in regulation of the development of CRA and metabolic disorders. Anyhow, regulation of miR-34a-mediated potential metabolic regulators such as *KLF4* may represent a therapeutic scheme for CRA, especially CRA complicated with metabolic disorders.

Besides these key molecules which were identified by constructing regulatory networks, some of other differentially expressed molecules that regulated the development of CRA and metabolic disorders have been reported. The upregulated genes *MMP7*, *LGR5*, and *CD44* in our study when comparing the CRA samples with normal ones have already been shown to be overexpressed in CRA (Kirimlioglu et al., 2006; Rho et al., 2018; Yang et al., 2018). These decreased expression of genes could be induced by metformin, which have been proved in a variety of tumors including colorectal tumors, ultimately inhibiting tumor growth (Yu et al., 2015; Mogavero et al., 2017). Another gene *SCD* which was upregulated in our study when comparing the CRA samples with normal ones, could be suppressed by metformin, subsequently leading to the decrease of fat mass and increase of insulin sensitivity (Kim et al., 2011). Pickens et al. (2016) demonstrated that *SCD* was significantly associated with adenomas. The gene *PRKAA2* that encodesα2 catalytic domains of adenosine monophosphate activated protein kinase (AMPK), was downregulated when comparing the CRA samples with normal ones in our study. It has been widely reported that the AMPK/mTOR signaling pathway was associated with metabolic disorders and tumorigenesis (Kim et al., 2011; Yu et al., 2015; Pickens et al., 2016; Mogavero et al., 2017). These studies have additionally suggested that activation of AMPK and suppression of the mTOR pathway could be induced by metformin, which may partly explain one of a class of hypoglycemic agent known as metformin has the potential to treat and prevent cancers. *GCG*, one of genes with high degrees in PPI network, was downregulated when comparing the CRA samples with normal ones in our study. Past study has shown that *GCG* was constantly downregulated in the colorectal normal mucosa-adenoma-carcinoma sequence (Wu et al., 2018). The expression of *GCG* could be upregulated by metformin in the gastrointestinal tract, subsequently elevating the plasma concentrations of GLP-1 that could provide hypoglycemic effect of exogenously for patients with type 2 diabetes (Migoya et al., 2010). In addition, some of DEMs, which were identified when comparing the CRA samples with normal ones in this study, were reported to play an important role in metformin-mediated anti-tumor effect. miR-26a and miR-101 were downregulated in our study when comparing the CRA samples with normal ones. Metformin could cause reexpression of these two miRNAs, subsequently causing cancer stem cell (CSC)-related anti-tumor effects (Bao et al., 2012). The agent also affected the expression of a CSC marker *CD44* that was upregulated in our study (Bao et al., 2012; Mogavero et al., 2017). These results suggested that metformin might exert its anti-tumor effect by targeting CSC-related pathway besides AMPK/mTOR signaling pathway. Indeed, some clinical studies to assess chemopreventive effects of metformin on CRA have been carried out. A phase 3 clinical study demonstrated that significant CRA reduction was achieved by the administration of low-dose metformin in patients without diabetes (Higurashi et al., 2016). However, another phase IIa trial reported that metformin did not reduce rectal mucosa pS6 or Ki-67 levels in obese patients with adenomas (Zell et al., 2020). These seemingly contradictory results suggested the complexity of mechanisms underlying metformin treatment and prevention for CRA. These differentially expressed molecules involving different signaling pathways, which may be as potential targets for the treatment and prevention of CRA, remaining to be further studied in CRA patients with different conditions. For further understanding of miRNA function, the miRNA-target gene-TF regulatory networks for these key miRNAs were constructed. MiR-34a and two TFs such as SOX10 and CNOT4 were identified as co-regulators of *AHCYL2*, *PDGFRA* and *MGAT4A*. MiR-96 and HDAC2 were identified as co-regulators of *RDX*, *MAF* and *SCN9A*. MiR-29c and five TFs such as IRX5, TEAD4, MAFK, NFIC and PBX3 were identified as co-regulators of *COL5A1.* However, in view of the complexity of miRNA and TF crosstalk, we need to look very carefully at these regulatory relationships. In any case, these potential regulatory patterns may throw light on discovering new molecular targets for CRA diagnosis and treatment. More investigations and validation *in vitro* and vivo are needed to elucidate the roles of these novel markers in CRA in the future.

## Conclusion

In conclusion, our study identified 514 DEGs and 167 DEMs between CRA tissues and normal ones. A total of 80 overlaps were screened among these DEGs and target genes of DEMs. Two key DEMs such as hsa-miR-34a and hsa-miR-96 were identified. In addition, another key DEM hsa-miR-29c, which was identified when we compared the differentially expressed molecules found in the comparison CRA versus normal samples to the ones obtained in the comparison CRC versus CRA, was also identified in CRC samples compared to CRA ones. Besides, network analysis revealed the co-regulatory associations among these key miRNAs, corresponding targeted genes and TFs in CRA. Nevertheless, the present study has some limitations. On the one hand, the sample size of most microarray data is small, especially the sample size of miRNA profile dataset. Thus, future research with larger samples is very necessary. On the other hand, we do not validate experimentally the expression of the aforementioned key genes, miRNAs and TFs. Notably, in consideration of the complexity of miRNA and TF crosstalks, these regulatory relationships need to be taken with caution. the roles of these key molecules in CRA and the mechanism of miRNA-target gene-TF regulatory networks need more investigations and validation *in vitro* and *vivo*.

## Data Availability Statement

The datasets of GSE31905, GSE4183, GSE37364, GSE41657, and GSE41655 were downloaded from the GEO database.

## Author Contributions

YG and JQ contributed to the conception of the study. YG and SZ contributed significantly to analysis and manuscript preparation. YG and YZ performed the data analyses and wrote the manuscript. JQ helped perform the analysis with constructive discussions.

## Conflict of Interest

The authors declare that the research was conducted in the absence of any commercial or financial relationships that could be construed as a potential conflict of interest.

## Footnotes

1 http://www.ncbi.nlm.nih.gov/geo/geo2r/

2 http://string-db.org/

## Supplementary Material

FIGURE S1 The workflow of the methodology used in the study. miRNA(s), micro-RNA(s); TF(s), transcription factor(s); CRA, colorectal adenoma; CRC, colorectal cancer; DEGs_a_, differentially expressed genes found in the comparison CRA samples versus normal samples; DEMs_a_, differentially expressed miRNA found in the comparison CRA samples versus normal samples; DEGs_b_, differentially expressed genes found in the comparison CRC samples versus CRA samples; DEMs_b_, differentially expressed miRNA found in the comparison CRC samples versus CRA samples.

FIGURE S2 Protein–protein interaction (PPI) network conducted based on 514 DEGs between CRA and normal tissues. Red nodes and green nodes indicate upregulated and downregulated genes, respectively. Diamond nodes represent the identified hub genes. PPI, protein-protein interaction; DEGs, differentially expressed genes; CRA, colorectal adenoma.

FIGURE S3 Regulatory network of DEMs and their target DEGs in the comparison CRC samples versus CRA samples. Red nodes and green nodes indicate upregulated genes/miRNAs and downregulated genes/miRNAs, respectively. Triangles represent DEMs and circles represent target DEGs. miRNAs, microRNAs; DEMs, differentially expressed miRNAs; DEGs, differentially expressed genes; CRA, colorectal adenoma; CRC, colorectal cancer.

TABLE S1 Differentially expressed genes between CRA tissues and normal tissues identified from GSE31905, GSE4183, GSE37364, and GSE41657. CRA, colorectal adenoma.

TABLE S2 Regulatory networks of the key miRNAs, target genes and transcription factors. miRNAs, microRNAs.
