# Supplementary figures and images for: Identification of MicroRNA-Target Gene-Transcription Factor Regulatory Networks in Colorectal Adenoma Using Microarray Expression Data
Source: Front Genet. 2020 May 19;11:463. doi: 10.3389/fgene.2020.00463 (PMC7248367; doi:10.3389/fgene.2020.00463)

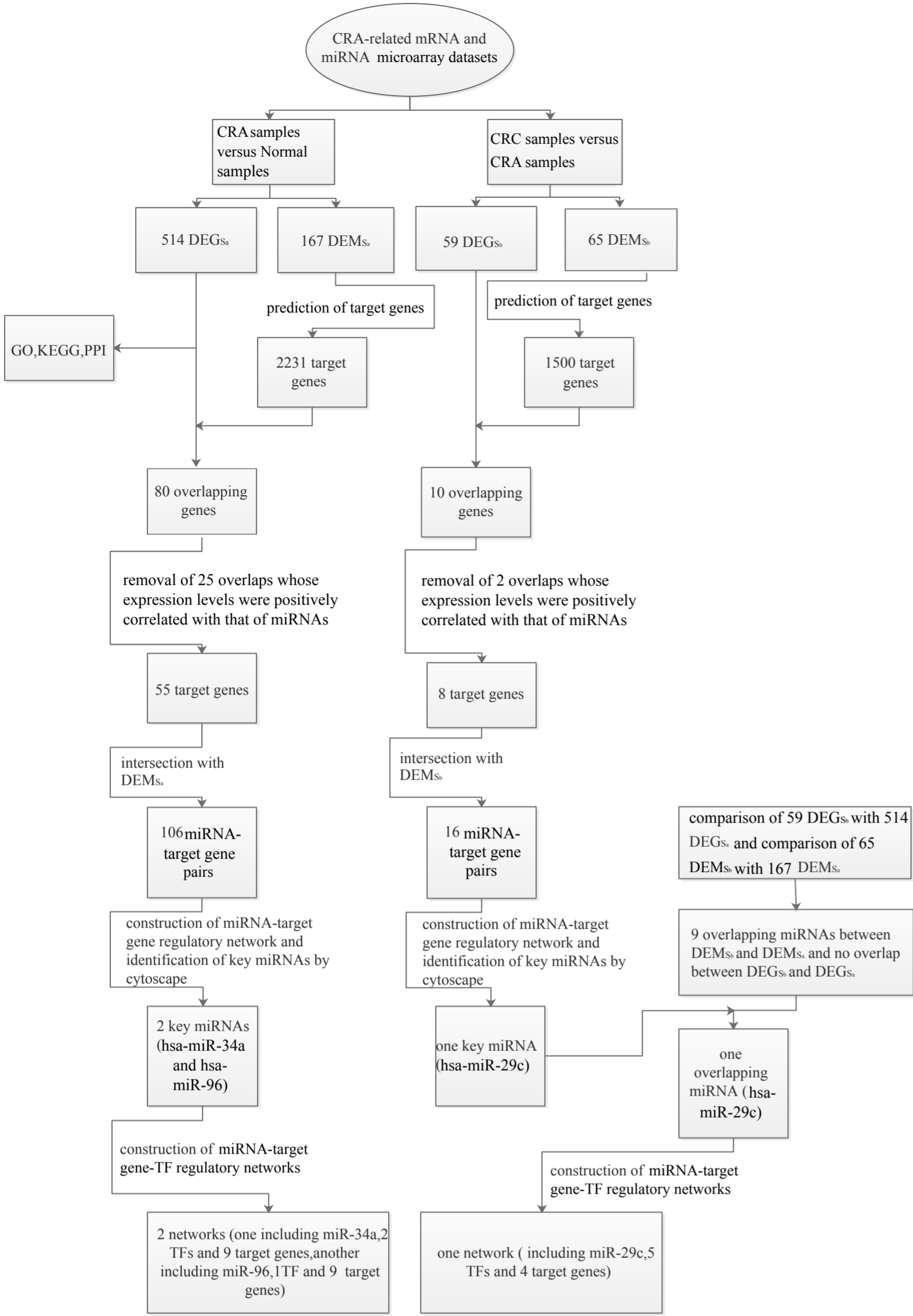

Supplement: FIGURE S1 — The workflow of the methodology used in the study. miRNA(s), micro-RNA(s); TF(s), transcription factor(s); CRA, colorectal adenoma; CRC, colorectal cancer; DEGsa, differentially expressed genes found in the comparison CRA samples versus normal samples; DEMsa, differentially expressed miRNA found in the comparison CRA samples versus normal samples; DEGsb, differentially expressed genes found in the comparison CRC samples versus CRA samples; DEMsb, differentially expressed miRNA found in the comparison CRC samples versus CRA samples. [file Image_1.pdf]

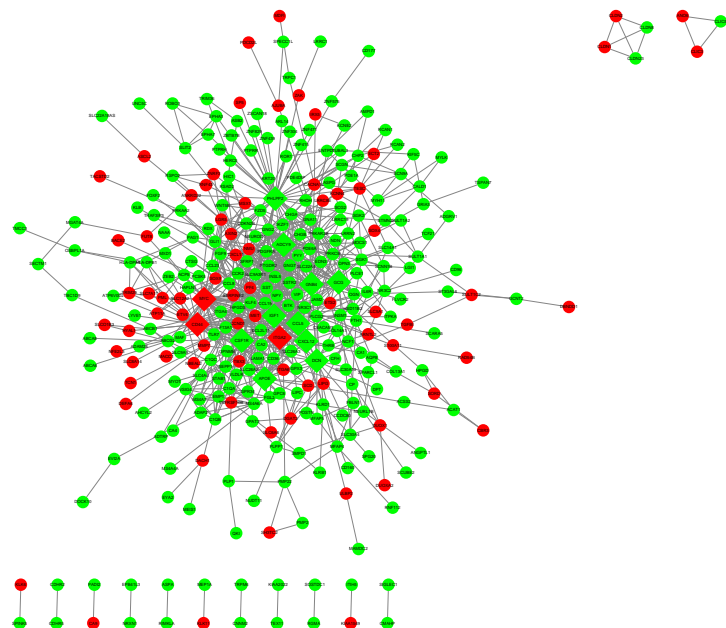

Supplement: FIGURE S2 — Protein–protein interaction (PPI) network conducted based on 514 DEGs between CRA and normal tissues. Red nodes and green nodes indicate upregulated and downregulated genes, respectively. Diamond nodes represent the identified hub genes. PPI, protein-protein interaction; DEGs, differentially expressed genes; CRA, colorectal adenoma. [file Image_2.pdf]

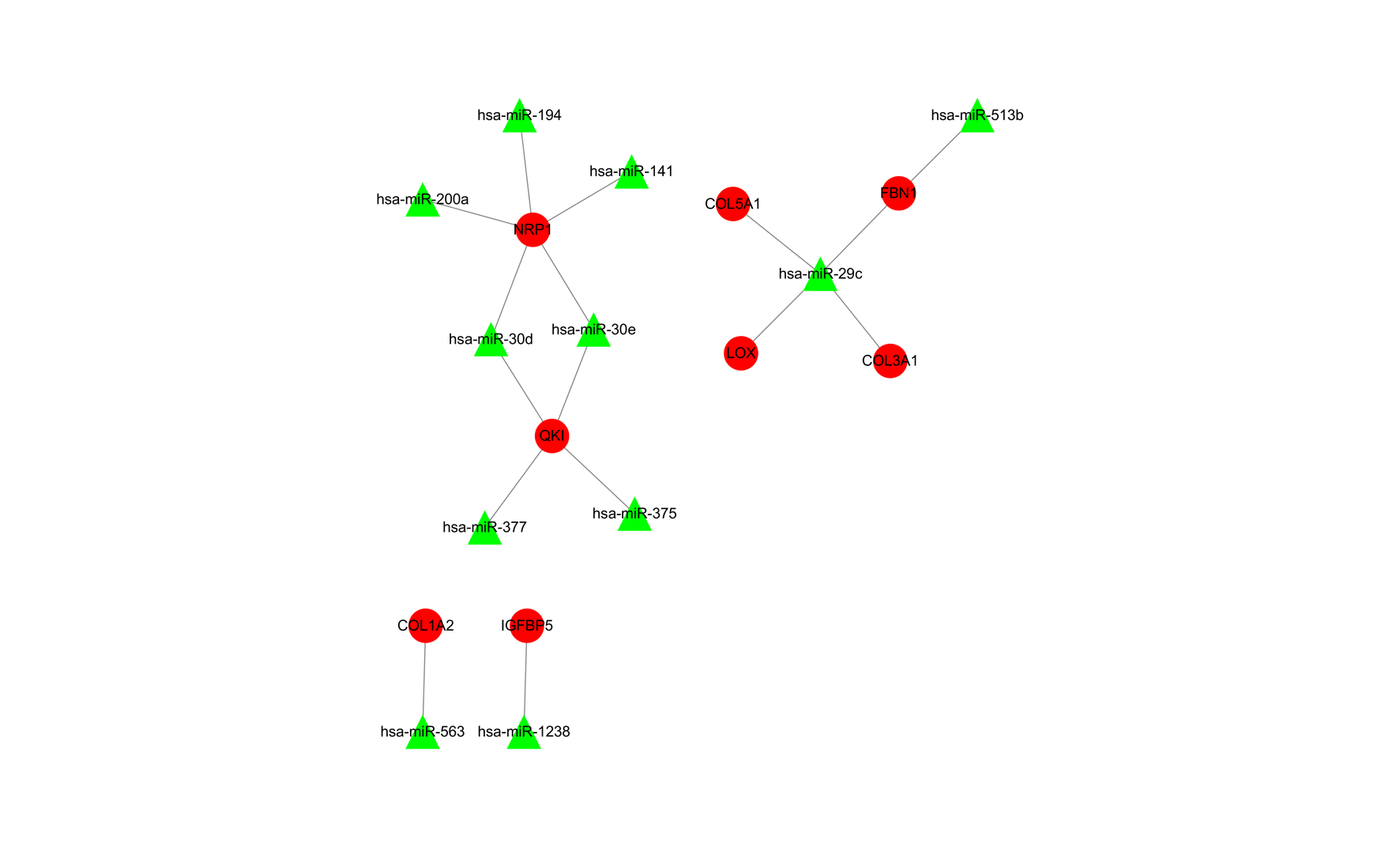

Supplement: FIGURE S3 — Regulatory network of DEMs and their target DEGs in the comparison CRC samples versus CRA samples. Red nodes and green nodes indicate upregulated genes/miRNAs and downregulated genes/miRNAs, respectively. Triangles represent DEMs and circles represent target DEGs. miRNAs, microRNAs; DEMs, differentially expressed miRNAs; DEGs, differentially expressed genes; CRA, colorectal adenoma; CRC, colorectal cancer. [file Image_3.tif]
